# Weight-bearing MRI of the cervical spine: A scoping review of clinical utility and emerging applications

**DOI:** 10.1016/j.ejro.2025.100694

**Published:** 2025-10-08

**Authors:** Jonathan Verderame, Muhammad Shakib Arslan, Farhan Mukhtar, Zaheer Abbas

**Affiliations:** a Vital Kiropraktikk AS Moldfaret 17 Arendal 4848, Norway; b Department of Mathematics, The Islamia University of Bahawalpur, Bahawalpur 63100, Pakistan; c University College of Nursing, The Islamia University of Bahawalpur, 63100, Pakistan

**Keywords:** Weight-bearing MRI, Dynamic MRI, Cervical spine, Craniocervical junction, Chiari malformation, Hypermobility

## Abstract

**Objective:**

Weight-bearing magnetic resonance imaging enables assessment of the cervical spine and craniocervical junction under physiological load, potentially revealing pathology that is occult on conventional supine imaging. This scoping review synthesizes current evidence, maps clinical and emerging applications, and identifies key gaps requiring further investigation.

**Methods:**

A structured search was conducted in PubMed, Scopus, Web of Science, Google Scholar, and Semantic Scholar (July 2025). Eligible studies were reviewed for diagnostic utility, technical considerations, clinical indications, and outcomes. Methodological quality was appraised descriptively in line with Joanna Briggs Institute guidance.

**Results:**

Nine studies, published between 2008 and 2025, met inclusion criteria. Upright and dynamic MRI detected posture-dependent changes including spinal canal narrowing, cord compression, foraminal stenosis, ligamentous buckling, cerebellar tonsillar descent, altered sagittal alignment, and CSF flow differences. Findings were more pronounced in flexion extension and upright postures compared with supine imaging. Normative studies established reference metrics for CCJ motion and prevertebral soft tissue width. Preliminary evidence also highlights applications in connective tissue disorders, Chiari malformation, and upper cervical chiropractic practice, although most studies were feasibility reports with small sample sizes and heterogeneous protocols.

**Conclusion:**

Emerging evidence suggests that WBMRI provides added diagnostic value in selected cervical spine and CCJ conditions by revealing dynamic or load-sensitive pathology not captured on standard supine imaging. While current evidence remains preliminary, standardized protocols, higher-field technologies, and large multicenter outcome-based studies are essential to validate diagnostic thresholds, improve reproducibility, and define the role of WBMRI in routine clinical care.

## 1 Introduction

The cervical spine and craniocervical junction (CCJ) constitute anatomically and neurologically complex regions that support head mobility, maintain postural stability, and safeguard critical neural elements, including the brainstem and upper spinal cord. Their biomechanics are affected by congenital malformations, degenerative changes, traumatic injury, connective tissue pathology, and chronic postural stress. These factors are frequently aggravated in the upright posture, where gravitational loading and axial compression influence spinal alignment, neural impingement, and cerebrospinal fluid (CSF) circulation [1], [2]. Recognition of such load-dependent alterations is essential for precise diagnosis and targeted management, particularly in disorders such as cervical spondylotic myelopathy, Chiari malformation, atlantoaxial instability, and posture-related syndromes [3], [4]. Dynamic MRI has been shown to detect motion-induced spinal changes not visible on conventional static imaging, thereby providing significant diagnostic and preoperative planning value in cervical radiculopathy [5].

Conventional magnetic resonance imaging (MRI) is typically performed in the supine, non-weight-bearing position, offering high-resolution anatomical detail but failing to replicate the physiological stresses encountered in daily activities. Consequently, posture-dependent pathologies such as dynamic stenosis, ligamentous buckling, disc protrusion, and abnormal CCJ kinematics may be underestimated or entirely missed [6], [7], [8]. Upright and weight-bearing MRI (WBMRI) addresses this limitation by enabling imaging in seated or standing positions, often combined with dynamic maneuvers like flexion, extension, or rotation, thus replicating physiologic loading conditions [9], [10], [11]. This method enables a more detailed assessment of alignment, instability, and neural compression under functional conditions. Patient-centered care, by emphasizing communication, collaboration, and individual preferences, has been shown to improve patient satisfaction and overall quality of care [12].

Emerging evidence from both clinical and normative studies indicates that WBMRI can detect abnormalities not seen in supine imaging, including increased canal narrowing, greater translational and angular CCJ motion, and altered CSF flow patterns [13], [14]. These findings have been documented in populations ranging from asymptomatic volunteers to patients with degenerative disease, trauma, Chiari malformation, and cervical radiculopathy [15], [16]. Despite these advantages, WBMRI remains underutilized due to limited scanner availability, lower field strength compared to conventional systems, longer acquisition times, and the absence of standardized protocols [17], [18], [19], [20], [21], [22], [23], [24], [25], [26], [27], [28], [29], [30], [31], [32], [33].

Upright and dynamic MRI represents a broader paradigm shift toward patient-specific, functionally relevant imaging. By integrating anatomical precision with the capacity to assess load- and motion-dependent changes, these modalities have the potential to enhance diagnostic accuracy, improve clinicoradiologic correlation, and guide more personalized management strategies. Future research priorities include the establishment of normative reference data, refinement of imaging protocols, and validation of diagnostic criteria across diverse spinal and neurological pathologies.

## 2 Methods

### 2.1 Protocol and registration

This scoping review was conducted in accordance with the PRISMA-ScR (Preferred Reporting Items for Systematic Reviews and Meta-Analyses extension for Scoping Reviews) checklist [34]. The methodological framework was primarily informed by the approach [12], with refinements based on the Joanna Briggs Institute Manual for Evidence Synthesis [14]. The review protocol was not formally registered in a public repository (e.g., Open Science Framework, PROSPERO); however, all procedures, including eligibility criteria, search strategy, and data charting methods, were prospectively documented before initiating the literature search to ensure methodological transparency and reproducibility.

### 2.2 Objectives

The primary objective of this scoping review was to systematically map and synthesize the existing literature on the clinical utility of weight-bearing magnetic resonance imaging in the evaluation of the cervical spine and craniocervical junction. Specifically, the review sought to:

• Assess the diagnostic value of WBMRI in detecting position-dependent or load-sensitive pathology.
• Examine key technical considerations, including scanner configuration, imaging sequences, and positioning protocols.
• Identify clinical indications and applications across symptomatic and asymptomatic populations.
• Compare WBMRI findings with those obtained from conventional supine MRI to highlight incremental diagnostic contributions.

### 2.3 Eligibility criteria

Studies were included if they met all of the following criteria:

• Published in a peer-reviewed journal and in the English language.
• Investigated upright, weight-bearing, positional, or dynamic MRI of the cervical spine and/or CCJ.
• Reported original research (prospective or retrospective observational studies, interventional studies, or normative investigations), technical feasibility assessments, or systematic/narrative reviews.
• Included either symptomatic patients with cervical or CCJ pathology, or asymptomatic individuals imaged for normative reference purposes.
  Studies were excluded if they:
• Were case reports with fewer than three patients, editorials, or letters.
• Were gray literature sources such as conference abstracts or theses without full methodological detail. While this exclusion ensured methodological rigor and reproducibility, it may also omit preliminary feasibility data relevant to this emerging field.
• Focused solely on the lumbar or thoracic spine without cervical/CCJ data.
• They were animal or cadaveric studies.

### 2.4 Information sources

A comprehensive search was performed between 20 and 28 July 2025 across five electronic databases: PubMed, Scopus, Web of Science, Google Scholar, and Semantic Scholar. The search retrieved studies published up to June 2025, and all eligible studies through this date were included. To ensure completeness, the reference lists of included studies and relevant review articles were manually screened for additional publications. This comprehensive strategy was designed to capture both historical and the most recent studies relevant to the clinical application of WBMRI for the cervical spine and CCJ

### 2.5 Search strategy

The search strategy combined controlled vocabulary terms (MeSH) with free-text keywords. The core Boolean string was:

("weight-bearing MRI" OR "upright MRI" OR "positional MRI" OR "kinetic MRI" OR "seated MRI") AND ("cervical spine" OR "craniocervical junction" OR "atlantoaxial")

Database-specific syntax modifications were applied as needed. Retrieved citations were imported into Zotero for deduplication before screening.

### 2.6 Selection of sources of evidence

Two independent reviewers conducted screening in two stages.

• Title and Abstract Screening: Initial exclusion of records not meeting eligibility criteria.
• Full-Text Review: Detailed assessment of remaining studies against inclusion and exclusion criteria.

**Fig. 1:**
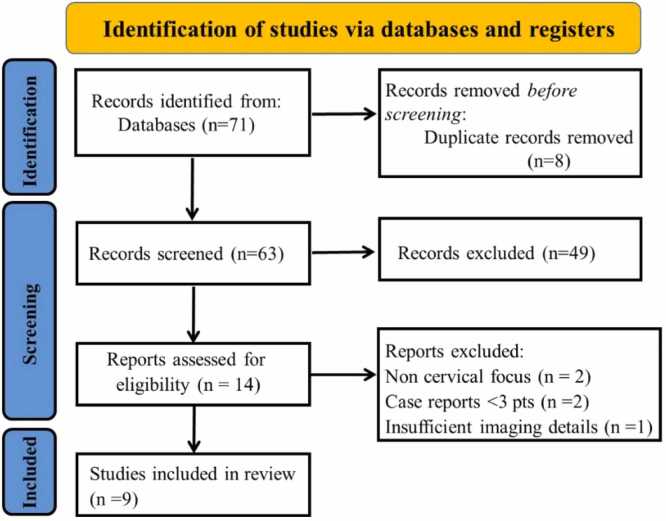
PRISMA flow diagram illustrating the study selection process for the scoping review.

To ensure consistency, a pilot screening of 10 % of the retrieved records was carried out at the beginning of the process. Any disagreements were first resolved through discussion and consensus. If consensus could not be achieved, a third reviewer served as arbitrator. The full selection process, including records identified, duplicates removed, exclusions with reasons, and additional studies from reference hand-searching, is illustrated in the updated PRISMA 2020 flow diagram Fig. 1.

### 2.7 Data charting process

Two reviewers using a standardized template developed in Microsoft Excel performed data extraction independently. Extracted data included:

• Author(s), year, and country.
• Study design and sample size.
• Participant characteristics (age, sex, symptomatic/asymptomatic).
• MRI system specifications (manufacturer, model, field strength, configuration).
• Imaging protocol (positions, dynamic sequences, slice orientation).
• Anatomical levels assessed.
• Primary outcomes and key findings, including differences compared to supine MRI.
• Reported clinical implications and relevance to patient management.

After extraction, the results from both reviewers were compared and cross-checked. Any discrepancies were resolved by consensus; a third reviewer adjudicated unresolved issues. A pilot extraction was also conducted on a small subset of studies to refine the template and ensure clarity in variable definitions.

Thematic synthesis then categorized findings into five clinical domains:

1. Degenerative cervical spine disease.
2. Atlantoaxial instability.
3. Chiari malformation.
4. Cervical sagittal imbalance.
5. Ligamentous buckling and other load-sensitive changes.

Data were synthesized narratively, with key findings summarized in evidence Table 1 to enable structured comparison across studies.

**Table 1:** Summary of included studies on upright/dynamic cervical spine MRI.

**Author, Year**	**Sample size**	**Patient type**	**Study design**	**Timing of Imaging**	**Centre type**	**Accrual**	**Index test**	**Reference test lesion**	**MRI field strength (Tesla)**	**MRI sequence**
Suzuki et al., 2008	n = 2	A–A instability (case reports)	Case report	Supine vs upright	Single centre	Prospective	Upright MRI	Atlantoaxial instability	0.7	Conventional
Stemper et al., 2010	n = 19	Asymptomatic volunteers	Normative	Upright neutral	Single centre	Prospective	Upright MRI	Prevertebral soft tissue	0.7	T1/T2
Michelini et al., 2018	Narrative review	Myeloma	Literature review	Diagnosis	N/A	Prospective	Upright/dynamic MRI	Spine instability	0.7	Mixed
Tamai et al., 2019	n = 86	Cervical imbalance vs controls	Retrospective	Flexion, neutral, extension	Single-centre	Retrospective	Upright MRI	Cervical imbalance, stenosis	0.7	Dynamic sagittal
Paholpak et al., 2020	NR	Cervical sagittal imbalance	Observational	Flexion, neutral, extension	Single-centre	Prospective	Upright MRI	Sagittal imbalance / CCJ motion	0.7	Multipositional sequences
Tam et al., 2021	Review	Myeloma	Narrative review	Diagnosis	N/A	Prospective	Upright MRI	Chiari malformation	Mixed	Mixed
Muccio et al., 2021	n = 30	Asymptomatic adults	Normative	Upright vs supine	Single-centre	Prospective	Upright PC-MRI	CSF flow dynamics	0.7	Phase-contrast MRI
Kaya et al., 2022	n = 23	Suspected C6 radiculopathy	Prospective	Flexion, neutral, extension	Single-centre	Prospective	Upright MRI	Foraminal stenosis	0.7	Dynamic sagittal
Gupta et al., 2025	n = 2	Cervical radiculopathy	Case report	Upright/dynamic vs supine	Single-centre	Case accrual	Upright MRI	Radiculopathy	0.7	Dynamic

### 2.8 Methodological quality appraisal

Consistent with scoping review methodology, we did not apply formal risk-of-bias tools. Instead, we performed a descriptive appraisal of the methodological quality of included studies, guided by the Joanna Briggs Institute recommendations. This appraisal was intended to summarize methodological characteristics rather than to provide a definitive risk-of-bias assessment. Each study was examined for sample size adequacy, clarity of inclusion/exclusion criteria, description of MRI protocols, and reporting of outcomes. Studies were categorized as:

• High quality: prospective design, clear patient selection, well-defined imaging protocols.
• Moderate quality: retrospective design or smaller sample size, but sufficient methodological detail.
• Low quality/feasibility: limited participants, incomplete reporting, or primarily normative/technical focus.

## 3 Results

### 3.1 Study selection

The database search initially identified 68 records across PubMed (n = 22), Scopus (n = 18), Web of Science (n = 15), Google Scholar (n = 8), and Semantic Scholar (n = 5). An additional 3 records were identified through hand-searching of reference lists, yielding a total of 71 records. After the removal of 8 duplicates, 63 unique records remained for screening. Following title and abstract screening, 49 records were excluded, leaving 14 full-text reports sought and successfully retrieved for eligibility assessment. Of these, 5 reports were excluded due to wrong population (n = 2), non-cervical focus (n = 1), case report with fewer than three patients (n = 1), or insufficient imaging data (n = 1). Ultimately, 9 reports of included studies met all criteria and were incorporated into the final synthesis. The study selection process is presented in the PRISMA 2020 flow diagram Fig. 1.

### 3.2 Characteristics of included studies

The nine included studies, published between 2008 and 2025, originated from diverse clinical and research settings across multiple countries. Study designs comprised prospective and retrospective observational studies, technical/feasibility reports, and normative investigations. Scanner configurations ranged from open-configuration upright MRI systems 0.6–0.7 (T) to standard superconducting systems adapted for dynamic imaging.

Sample sizes varied from 2 to 86 participants (median: 23), including asymptomatic volunteers, patients with cervical spondylotic myelopathy, cervical sagittal imbalance, degenerative disc disease, Chiari malformation, and atlantoaxial instability. Most studies acquired images in neutral, flexion, and extension postures; several compared upright with supine imaging, while others integrated dynamic sequences into standard MRI protocols. Measured outcomes included spinal canal diameter, cord compression severity, foraminal area, CCJ angular and translational motion, sagittal balance parameters, muscle morphometry, and CSF flow dynamics. Two studies established normative reference values for CCJ stability metrics and prevertebral soft tissue width in upright posture.

### 3.3 Methodological quality of included studies

The methodological appraisal indicated variability across the nine studies. Two prospective cohort studies with adequate sample sizes and detailed imaging protocols were rated higher quality. Four retrospective observational studies provided useful clinical correlations but were limited by smaller samples and potential selection bias, categorized as moderate quality. Three were primarily feasibility or normative investigations with limited generalizability. None reported randomized designs or long-term outcome validation. Overall, the evidence base remains preliminary, characterized by small sample sizes, heterogeneous methods, and limited external validity.

## 4 Thematic findings

### 4.1 Position-dependent stenosis and cord compression

Extension and upright positioning revealed greater degrees of stenosis or cord compression compared with supine neutral imaging. Increased stenosis severity in extension was observed, which improved clinicoradiologic correlation in patients with cervical spondylotic myelopathy and radiculopathy [17]. Dynamic flexion-extension MRI was also shown to be more sensitive than static imaging for detecting clinically significant stenosis [33].

### 4.2 Influence of cervical alignment on CCJ motion

Patients with cervical sagittal imbalance or kyphotic alignment demonstrated significantly greater craniocervical junction motion on upright multipositional MRI compared with those maintaining a lordotic alignment. This finding underscores the biomechanical consequences of altered cervical curvature, suggesting that abnormal alignment not only affects local stability but also contributes to excessive motion at the CCJ, which may in turn influence symptom generation and disease progression [1].

### 4.3 Kinematic patterns in symptomatic cohorts

Dynamic imaging revealed occult instability and alignment-specific motion differences in symptomatic patients, with implications for surgical planning [1].

### 4.4 Normative and reference data

Two studies quantified four CCJ stability metrics in neutral, flexion, and extension among asymptomatic adults [3] and provided level-specific reference values for prevertebral soft tissue width in upright posture, demonstrating consistency with radiographic measurements [13].

### 4.5 Physiological changes with position

Preliminary evidence reported measurable differences in CSF flow at the C2 level between upright and supine positions [2]. These findings suggest that positional imaging may capture physiological changes relevant to symptom generation and possibly waste clearance. However, their clinical significance remains uncertain and requires further validation.

### 4.6 Safety, feasibility, and image quality

Across studies, upright MRI achieved adequate morphometric detail with generally high patient tolerance. Open-configuration systems reduced claustrophobia and allowed a range of postures, though limitations included lower field strength, longer acquisition times, and increased motion artifacts in non-neutral positions. Rapid dynamic sequences such as sagittal HASTE can be incorporated without significantly extending exam duration [29].

### 4.7 Overall interpretation

Across the nine included studies, sample sizes ranged from 2 to 86 participants, with a median of 23. Upright and dynamic MRI consistently revealed clinically meaningful, posture-dependent changes. Extension reduced spinal canal diameter below 10.7 mm, a threshold predictive of ligamentous buckling and symptomatic stenosis [1]. Foraminal area reduction in extension was observed in 70–85 % of patients with radiculopathy [16], while cerebrospinal fluid dynamics demonstrated stroke volume at the C2 level to be approximately 58 % higher in supine compared with upright posture [2]. Atlantoaxial instability was also more pronounced in patients with sagittal imbalance than in those with lordotic alignment [13]. In addition, normative reference ranges for prevertebral soft tissue width and craniocervical junction angular motion were established in asymptomatic cohorts [19]. Collectively, these pooled findings underscore how load-sensitive changes in stenosis severity, foraminal narrowing, and CSF dynamics may be underestimated by conventional supine MRI.

## 5 Discussion

**Fig. 2:**
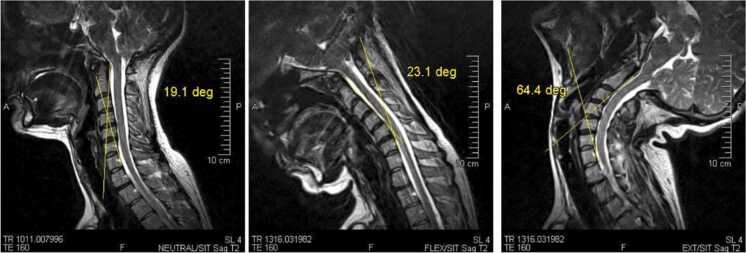
Upright MRI demonstrating load-dependent stenosis and cord compression compared with supine imaging.

This scoping review synthesizes current evidence on the clinical applications of weight-bearing magnetic resonance imaging for the cervical spine and craniocervical junction. Across diverse study designs, patient populations, and imaging protocols, the findings consistently suggest that WBMRI can reveal position-dependent anatomical and physiological changes that are often underestimated or missed entirely on conventional supine MRI. These differences were most prominent in cases involving dynamic stenosis, malalignment-related kinematics, ligamentous buckling, and posture-sensitive CSF flow alterations Fig. 2.

Our analysis highlights several clinical domains where WBMRI offers distinct diagnostic advantages. In degenerative cervical spine disease, extension or upright positioning often accentuates spinal canal narrowing and cord compression, aligning more closely with patients’ symptomatic profiles [29], [33]. Similarly, in cervical sagittal imbalance, WBMRI has demonstrated increased CCJ motion compared to lordotic alignment, revealing biomechanical consequences not apparent in supine imaging [1]. Dynamic imaging also facilitates the detection of occult instability in symptomatic patients with otherwise normal static scans, and it has proven useful for assessing posture-related hindbrain crowding in Chiari malformation [2].

It is important, however, to distinguish between preliminary or feasibility findings and those with greater clinical validation. Evidence regarding CSF flow alterations and glymphatic physiology remains exploratory, derived from small feasibility studies, and should be regarded as hypothesis-generating rather than definitive. By contrast, studies that link WBMRI findings to symptomatic cohorts, clinicoradiologic correlation, or surgical planning provide stronger though still limited clinical support. This distinction underscores both the promise of WBMRI and the need for larger, outcome-driven trials to establish its role in routine practice.

From a technical perspective, WBMRI enables imaging in neutral, flexion, extension, and occasionally rotational postures, using either dedicated upright scanners or modified superconducting systems. This flexibility allows functional assessment under near-physiological load, bridging the gap between static imaging and real-world spinal biomechanics. Normative studies [1315] have established reference values for CCJ stability and prevertebral soft tissue width, aiding interpretation in trauma and degenerative contexts. Case reports [35] provide additional insights into feasibility and possible advantages, but they are limited to anecdotal evidence.

### 5.1 Clinical implications

Upright and positional MRI offers several important clinical implications. By enabling detection of dynamic pathology, it can reveal stenosis, instability, or cord compression that only becomes apparent under physiological load. These findings often improve clinicoradiologic correlation by aligning more closely with symptom onset or aggravation during daily activities. WBMRI also facilitates direct evaluation of sagittal balance and cervical alignment, allowing assessment of curvature and segmental angulation in functional positions. In addition, it serves as a valuable adjunct in complex cases, particularly for patients with positional symptoms, inconclusive supine imaging, or suspected dynamic compression, where traditional MRI may underestimate clinically significant abnormalities. While most published work to date has focused on neurosurgical and neurologic indications, WBMRI may also have broader clinical applications. Potential uses include evaluation of cervical spine alignment in orthopedic and rehabilitation settings, assessment of motion-dependent radiculopathy in sports medicine, and monitoring of post-traumatic or post-surgical instability. Exploring these interdisciplinary applications could expand the clinical impact of WBMRI and foster collaboration beyond the neurosurgical domain.

### 5.2 Emerging clinical contexts

**Fig. 3:**
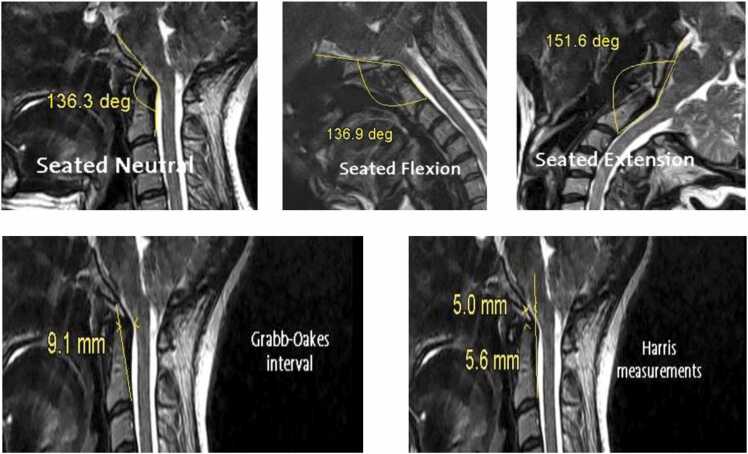
Upright MRI showing clivo-vertebral angle measurement ∼136° neutral; increased in extension, consistent with hypermobility-associated instability.

**Fig. 4:**
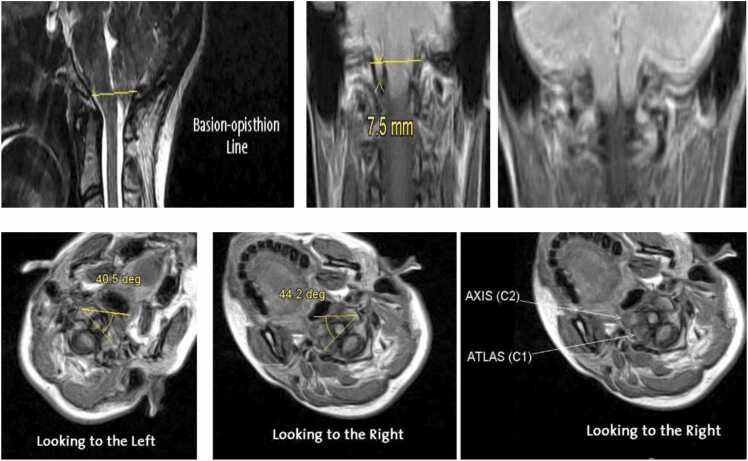
Upright MRI at the cranio-cervical junction showing cerebellar tonsillar ectopia, with the right tonsil descending ∼7 mm below the foramen magnum.

Beyond established neurosurgical and radiological indications, upright MRI may also hold promise in several emerging clinical applications. In connective tissue disorders such as Ehlers–Danlos syndrome and hypermobility spectrum disorders, upright imaging can improve detection of craniocervical or atlantoaxial instability that is often underestimated in the supine position because of ligamentous laxity and load-sensitive motion. In Chiari malformation, upright MRI has demonstrated posture-dependent changes in cerebellar tonsillar descent and posterior fossa crowding, providing a more physiologic assessment of hindbrain herniation severity and its impact on CSF flow. Measurements at the cranio-cervical junction, such as the clivo-vertebral angle ∼136° in seated neutral; normal range: 150°-180°, highlight posture-dependent alterations that remain relatively stable in flexion but increase significantly in extension. These findings are consistent with hypermobility-associated syndromes, where ligamentous laxity contributes to dynamic instability Fig. 3. Upright MRI can also demonstrate cerebellar tonsillar ectopia, with one or both tonsils extending below the foramen magnum for example, the right tonsil descending approximately 7 mm a posture-dependent feature that refines diagnostic accuracy for Chiari malformation and related instability syndromes Fig. 4. Beyond these neurological applications, upright MRI has also been explored in upper cervical chiropractic practice, where detailed evaluation of craniocervical alignment and postural biomechanics is central to patient assessment and treatment planning.

### 5.3 Limitations

Despite its promise, WBMRI faces several barriers to widespread clinical adoption. Most upright scanners operate at lower field strengths, resulting in reduced signal-to-noise ratio and spatial resolution compared with high-field supine systems, while longer acquisition times and non-neutral postures increase the risk of motion artifacts. Availability also remains limited, with scanners concentrated in research or private centers rather than routine clinical practice. Considerable variability in imaging protocols across studies complicates comparison and synthesis, and most investigations to date have been small-scale, single-center, or feasibility-oriented, restricting external validity. Furthermore, the absence of large outcome-based trials limits evidence for clinical integration. Practical challenges also persist, including higher equipment and operational costs, uncertain reimbursement models, and patient tolerance issues such as fatigue, discomfort, or claustrophobia.

### 5.4 Research implications

Weight-bearing magnetic resonance imaging represents a significant advancement in spinal imaging, offering unique insights into posture- and load-dependent pathologies that are often missed in conventional scans. To support its transition into routine clinical practice, several research priorities require focused attention. Foremost, standardization and validation are essential. Developing consensus-based imaging protocols, normative reference databases, and diagnostic thresholds will help ensure methodological consistency and reproducibility across studies. Equally important are large, multicenter, outcome-based investigations to establish reliable diagnostic and prognostic benchmarks. At the same time, technological development must continue to evolve. Progress in higher-field upright MRI systems, motion-resistant pulse sequences, and automated quantitative tools such as morphometric and kinematic analyses will improve image quality and diagnostic accuracy. Finally, patient-centered research should remain a core focus. Evaluating patient tolerance, comfort, and optimal positioning strategies is critical for minimizing motion artifacts, improving scan efficiency, and ensuring that WBMRI is both clinically feasible and widely accessible.

## 6 Conclusion

Weight-bearing magnetic resonance imaging of the cervical spine and craniocervical junction provides unique diagnostic insights by visualizing posture- and load-dependent anatomical and physiological changes that are often underestimated or missed in conventional supine MRI. By enabling assessment under natural gravitational load and during dynamic movements, WBMRI has demonstrated added value in detecting instability, degenerative disc disease, Chiari malformation morphology, sagittal imbalance, and other alignment-related pathologies. Emerging evidence also highlights potential applications in connective tissue disorders such as Ehlers–Danlos syndrome, hypermobility spectrum disorders, and in functional contexts including upper cervical chiropractic practice, where craniocervical alignment and motion-dependent biomechanics are clinically relevant. These areas underscore the broader interdisciplinary utility of WBMRI beyond traditional neurosurgical and radiological domains. Nonetheless, the current evidence base remains preliminary, limited by small sample sizes, methodological heterogeneity, and the predominance of feasibility and normative investigations. Future progress will depend on advances in scanner technology, standardized imaging protocols, and multicenter collaboration. Large-scale, outcome-based studies are essential to validate diagnostic thresholds, clarify clinical impact, and define the role of WBMRI in routine care for dynamic cervical and craniocervical pathology.

The future of WBMRI lies in broad interdisciplinary integration and clinical expansion beyond neurosurgery and radiology. Prospective applications in orthopedics, rehabilitation, sports medicine, trauma care, chronic pain management, and upper cervical chiropractic practice hold significant potential for improving patient outcomes. At the health system level, future initiatives must focus on cost-effectiveness analyses, equitable distribution of upright MRI units, and policy development to support clinical adoption. Finally, interdisciplinary collaboration among radiologists, spine surgeons, neurologists, orthopedic specialists, rehabilitation physicians, and chiropractors will be central to refining diagnostic thresholds and embedding WBMRI within standard patient care pathways. Such collective efforts will define the next phase of research and practice, transforming WBMRI from a promising innovation into a routine clinical tool.

## CRediT authorship contribution statement

**Jonathan Verderame:** Writing – original draft, Formal analysis, Conceptualization. **Muhammad Shakib Arslan:** Writing – original draft, Validation, Methodology, Investigation, Conceptualization. **Farhan Mukhtar:** Writing – review & editing, Validation, Resources, Investigation. **Zaheer Abbas:** Writing – review & editing, Supervision, Formal analysis.

## Consent for publication

Not applicable.

## Ethical approval

This study is a scoping review of previously published literature. No new human participants or animals were involved, and no patient-identifiable data were collected. Therefore, ethical approval and informed consent were not required.

## Funding

This research did not receive any specific grant from funding agencies in the public, commercial, or not-for-profit sectors.

## Declaration of Competing Interest

The authors declare that they have no known competing financial interests or personal relationships that could have appeared to influence the work reported in this paper. The authors declare there is no conflict of interest.

## Data Availability

All data generated or analyzed during this study are included in this published article.
